# Inter-Eye Comparison of the Ocular Surface of Glaucoma Patients Receiving Surgical and Medical Treatments

**DOI:** 10.3390/jcm11051238

**Published:** 2022-02-24

**Authors:** Dario Romano, Valentino De Ruvo, Paolo Fogagnolo, Roberta Farci, Luca Mario Rossetti

**Affiliations:** 1 Eye Clinic, ASST Santi Paolo e Carlo—San Paolo Hospital, University of Milan, 20143 Milan, Italy; dario.romano1@unimi.it (D.R.); valentino.deruvo@unimi.it (V.D.R.); luca.rossetti@unimi.it (L.M.R.); 2 Department of Health Science, University of Milan, 20146 Milan, Italy; roberta.farci@unimi.it

**Keywords:** glaucoma surgery, glaucoma medicaments, ocular surface, trabeculectomy, IDEEL, OSDI, Schirmer test, tear film osmolarity, tear break-up time, corneal staining

## Abstract

Background: Ocular surface frequently affects glaucoma patients. In this paper we aimed at evaluating the impact of glaucoma surgery on the ocular surface of patients who received unilateral trabeculectomy. Methods: 26 consecutive patients successfully treated with trabeculectomy on one eye (Trab Eye) and under control with topical treatments on the fellow eye (Med Eye) were included in this observational study. They received IDEEL and OSDI questionnaires, Tear Film Osmolarity (TFO), grading of conjunctival hyperemia, fluorescein tear break-up time (tBUT), grading of corneal staining and Schirmer test. Results: IDEEL and OSDI scores were 48 ± 38 and 11 ± 12, respectively, with moderate correlation (*r* = 0.50, *p* = 0.03). Compared with Med eyes, Trab Eyes had higher tBUT (6.5 ± 3.5 vs. 5.1 ± 2.7 s, *p* = 0.004), lower conjunctival hyperemia (0.8 ± 0.9 and 1.7 ± 1.1 respectively, *p* < 0.001) and lower corneal staining (0.3 ± 0.5 and 0.6 ± 0.5, respectively, *p* = 0.03). Correlation between corneal staining and conjunctival hyperemia was 0.55 in Trab Eyes (*p* = 0.01) and 0.44 in Med Eyes (*p* > 0.05). Patients with bilateral corneal staining had had threefold worse questionnaire scores (*p* < 0.05). The duration of treatment and the daily exposure to preservatives did not directly affect OS parameters in this cohort of patients. Conclusions: Patients receiving successful trabeculectomy showed better OS homeostasis (higher TBUT, lower grading of conjunctival hyperemia and corneal staining) than fellow medically treated eyes. Presence of corneal epithelial damage in both eyes is the factor more consistently affecting questionnaire scores.

## 1. Introduction

Glaucoma is the leading cause of irreversible blindness with more than 70 million people affected worldwide [1]. High intraocular pressure (IOP) is the only modifiable risk factor and its reduction is obtained by means of eye drops, which represent the first line of treatment, laser therapies, and surgery, in general considered as last option [2,3]. 

Both IOP-lowering eye drops and glaucoma surgery are frequently responsible for iatrogenic dry eye disease (DED), as they induce relevant changes to the ocular surface. More than 38% of glaucoma patients treated with just one eye drop have ocular surface disease, and the incidence increases with the number of medications used [4,5]. As a matter of fact, the International Dry Eye Workshop has classified IOP-lowering treatments as an extrinsic cause of evaporative dry eye [6].

Benzalkonium chloride (BAK) is the most used preservative in glaucoma eyedrops as it is effective in preventing microbial contamination. Still, it also breaks up the tight junctions increasing the space between epithelial cells; it increases oxidation due to mitochondrial depolarization, cytochrome c release and augmentation in caspases activity, which all negatively impact on the homeostasis of corneal epithelial cells, conjunctival goblet cells, and sub-basal nerve plexus [7]. It also reduces tear film stability by disrupting the lipid layer and reducing mucin secretion [8]. The clinical effects of BAK vs. BAK-free formulations have been explored by several papers [9,10,11,12]. Active compounds can also trigger ocular surface damage. Prostaglandin analogues (PGA) are pro-inflammatory molecules associated with tear production and stability, as well as epithelial damage [3,13]. Toxic effects have been also shown for preserved and nonpreserved beta blockers and α2-adrenergic agonists [14,15]. Finally, pH of different formulations may affect OS. A recent study showed that the use of brinzolamide-timolol suspension, with an almost neutral pH, is more comfortable than dorzolamide-timolol solution, which have an acid pH [16].

Successful glaucoma surgery may be associated with complete or partial suspension of IOP-lowering medications, hence ameliorating ocular surface homeostasis and reducing medicaments-related DED [17]. Nonetheless, surgery may induce changes for two main reasons: the use of mitomycin C can cause epithelial damage and goblet cell loss [18]; filtrating bleb also modifies the anatomy of the ocular surface and may induce DED by itself [19].

The purpose of this study was to analyze the ocular surface status between eyes treated medically and the fellow eyes treated surgically.

## 2. Materials and Methods

This observational clinical study was performed at the Eye Clinic of San Paolo Hospital, University of Milan. From September 2018 to September 2021, all patients attending the Glaucoma service and fulfilling inclusion criteria were asked to participate to this study. The research protocol was approved by the institutional review board (number of approval 16,043/2018, date: 28 March 2018) and carried out in accordance with the tenets of the Declaration of Helsinki. Written informed consent was obtained from each participant after full explanation of the aims of the study and its procedures.

Major inclusion criteria encompassed adults of both genders diagnosed with bilateral primary open-angle glaucoma (POAG), successfully treated with trabeculectomy on one eye and with medical treatment on the fellow eye. Only patients with complete success after trabeculectomy were included (IOP ranging between 5- and 18-mm Hg without antiglaucoma medications); only stable trabeculectomy cases were included (ie patients who were discharged from surgical follow-up and sent back to the medical service). Exclusion criteria were glaucomas other than POAG, neuropathic DED (due to diabetes, long-standing contact lens wearing, previous ocular herpes infections, previous eye surgery or other causes), Sjögren syndrome and/or other autoimmune disease, presence of corneal and ocular surface diseases other than DED (e.g. past or active cicatricial conjunctivitis, ocular surface burns, corneal trauma, keratinization of the eyelid margin and/or other functional and anatomic eyelid abnormalities), current use of contact lenses, pregnant and lactating women. Patients using lubricating eyedrops were instructed to stop them one week before the study. 

Trabeculectomy was performed using intraoperative 0.2 mg/mL mitomycin for 2–3 min. In total 7 patients received postoperative needling of the bleb + subconjunctival injection of 0.1 mL 5-fluoruracil (one case after 1 month from surgery, two cases after 2 months, three cases at 1 year and one case after 2 years); in all cases, study procedures were performed at least 1 year after this procedure. 

The medical history of POAG patients monitored at the Glaucoma Unit of our hospital were evaluated, and patients fulfilling inclusion and exclusion criteria were contacted to participate to this study. 

Enrolled patients underwent the following tests in the following order:

1. OSDI questionnaire (OSDI, Copyright 1995, Allergan Inc, Irvine, CA, USA);
2. Impact of Dry Eye on Everyday Life (IDEEL) questionnaire;
3. Tear Film Osmolarity (TFO) to both eyes;
4. anterior segment assessment at slit lamp to both eyes;
5. grading of conjunctival hyperemia to both eyes;
6. fluorescein tear break-up time (tBUT) to both eyes;
7. grading of corneal fluorescein staining to both eyes;
8. Schirmer test to both eyes.

One trained ophthalmologist performed the examinations during the whole study.

### 2.1. Study Procedures

OSDI questionnaire includes 12 questions to assess dry eye symptoms, which are subdivided into three groups (ocular symptoms, ability to perform daily activities; susceptibility to environmental factors). OSDI score ranges from 0 to 100 (0–12, normal; 13–22, mild DED; 23–32, moderate DED; 33 or more, severe DED) [20]. 

IDEEL questionnaire consists of 57 questions subdivided into three different modules focused on dry eye impact on daily life, dry eye treatment satisfaction and problems related with dry eye. Each module is scored from 0 to 100: higher scores in the first two modules indicate better QoL, and more severe DED in the third module [21].

TFO was measured by means of i-Pen device (I-MED Pharma Inc., Dollard-des-Ormeaux, QB, Canada). Tear sample was collected from the lower eyelid tear meniscus, while the patient fixating upwards. Care was paid to avoid contact between the probe and the globe [22,23]. TFO was measured at least one hour after the latest eye drops instillation. 

The bulbar conjunctival hyperemia was scored as follows: 0 (none: no hyperemia in the bulbar conjunctiva), 1 (mild: dilation of a few conjunctival blood vessels (2 or 3)), 2 (moderate: dilation of many conjunctival blood vessels (≤4 to 9 vessels)) or 3 (severe: dilation of all conjunctival blood vessels (≥10 vessels) [24].

TBUT was measured by determining the timeframe between end of blinking and tear break-up. TBUT was performed after diluting two strips on 0.5 mL of saline and instilling 5 microliters with the pipette 5% preservative-free sodium fluorescein solution into the inferior conjunctival tarsus. The dark spaces in the fluorescent tear film (seen at the slit lamp at 10× magnification using cobalt blue illumination) were indicative of tear break-up. These spaces should be confirmed by two consecutive inspections [25]. TBUT was measured with a stopwatch.

Corneal staining was measured using the Oxford scale after instillation of a fluorescein drop using the blue cobalt filter on the slit-lamp illumination. The Oxford system uses a scale consisting of a series six grades, labelled A–E in order of increasing severity. In each chart, staining is represented by punctate dots. To grade staining, comparisons are made between the panels and the appearance of staining on the exposed interpalpebral conjunctiva and cornea of the patient [26].

Schirmer test was performed without anesthesia, 15 min after corneal fluorescein test, in a dimly lit room. While the patient looked upwards a sterile strip was inserted into the conjunctival sac over the temporal one-third of the lower eyelid margin. After 5 min the strip was removed and the length of the tear absorption was measured [25].

### 2.2. Statistical Analysis

Sample size was calculated using the following formula:

(1) $$N=2*{\left[z_{\left(1-\alpha /2\right)}\sqrt{2\bar{p}\left(1-\bar{p}\right)}+z_{\left(1-\beta \right)}\sqrt{p_{1}\left(1-p_{1}\right)+p_{2}\left(1-p_{2}\right)}\right]}^{2}/\Delta^{2}$$

We assumed conjunctival hyperemia as a primary outcome. Hyperemia is present in nearly all medically treated eyes (*p*_1_ = 0.95), and surgery is frequently associated with hyperemia reduction; we assumed a 40% reduction of hyperemia as clinically relevant (*p*_2_ = 0.55) [27,28,29]. With α/2 = 0.05 and β = 0.8, Δ = 0.40, 25 patients were necessary in this study. 

Data were tested for normality using Shapiro–Wilk test and normality was confirmed. Data from both eyes were compared by means of t-test for paired data (continuous variables) and chi squared test (ordinal variables). Correlation between questionnaires was calculated using Pearson’s correlations (continuous variables) and Spearman test (ordinal variables). Statistical significancy was set at *p* < 0.05.

## 3. Results

In total, 26 consecutive patients with an history of trabeculectomy with complete success on one eye and receiving IOP-lowering treatments just on the fellow eye were included in this study. A total of 18 patients were males, age was 70.7 ± 12.3 years; 24 subjects were Caucasian, one Hispanic, one African. Surgery was performed on 13 right eyes and 13 left eyes, 31 ± 39 months (range 6–190 months) before study conduction. Duration of medical treatment on the fellow eye was 34 ± 34 months (range 8–186 months). The distribution of IOP-lowering treatments on the fellow eye are reported in Table 1. All patients except one (the one using tafluprost) were using at least one preserved medication. 10 patients were using lubricating eyedrops to both eyes, and they stopped them before undergoing clinical examination, and interestingly, these patients had similar ocular parameters compared with patients not using them (in this subgroup tBUT was 5.1 ± 2.1 and 6.6 ± 3.0 s in Med and Trab Eye respectively, Schirmer 13 ± 7 and 14 ± 8 mm/5 min, epithelial damage was present in 6/10 patients, and hyperemia in 7/10 Med Eyes and 3/10 Trab Eyes); OSDI was similar (11 ± 9) whereas IDEEL score was significantly higher at 65 ± 52 (*p* = 0.03).

The main results of the study are reported in Table 2. Comparing Trab Eyes and Med Eyes, a statistically significant difference was found for hyperemia (*p* < 0.001), tBUT (*p* = 0.004) and corneal staining (*p* = 0.03), with Trab Eyes showing better outcomes for both parameters. Inter-eye differences were negligible for Schirmer test and TFO.

For each patient, we evaluated whether each parameter was better in the Trab or Med eye; the distribution is reported in Table 3.

**Figure 1:**
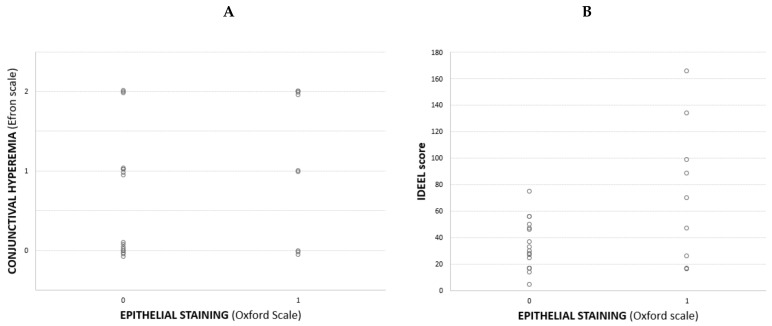
(**A**). Scatterplot of epithelial staining vs. conjunctival hyperemia in eyes receiving trabeculectomy (*r* = 0.55, *p* = 0.01); (**B**). Scatterplot of epithelial staining IDEEL, Impact of Dry Eye on Everyday Life score in eyes receiving trabeculectomy (*r* = 0.37, *p* = 0.06).

Correlations between study variables were calculated. IDEEL and OSDI questionnaires showed a moderate correlation of 0.56 (*p* = 0.02). In Med Eyes, significant correlations were found between Schirmer and TFO (*r* = 0.48, *p* = 0.04), and hyperemia vs. staining (chi-square 0.68). In Trab Eyes, significant correlations were found just between hyperemia and staining (*r* = 0.55, *p* = 0.01, Spearman test, Figure 1a); borderline data were found between IDEEL score and epithelial staining (*r* = 0.37, *p* = 0.06, Spearman test, Figure 1b). The other variables showed negligible correlations.

The postoperative exposure to 5-fluoruracil had no effect on ocular surface parameters (tBUT was 6.5 ± 4.2 s, Schirmer 10 ± 7 mm/5 min, IDEEL 35 ± 18, OSDI 8 ± 8, epithelial damage was present in 2/7 patients and hyperemia in 2/7 patients).

As epithelial staining was the parameter showing highest correlation with questionnaires, it was studied more in details. We divided the study cohort on three groups depending on the presence of epithelial defect: bilateral staining (*n* = 10), unilateral staining (*n* = 9), and no staining (*n* = 7). IDEEL score was 90 ± 42 in patients with bilateral staining; 33 ± 12 in unilateral staining, and 31 ± 13 in patients with no staining (*p* < 0.05 comparing bilateral staining vs. the other two groups). Interestingly, in unilateral staining, those with staining in Trab Eyes (*n* = 2) had lower IDEEL score than those on Med Eyes (*n* = 7) (21 ± 6 vs. 35 ± 20), though the difference was not significant at *p* = 0.07. OSDI score was 17 ± 10 in patients with bilateral staining; 6 ± 3 in patients with unilateral staining, and 5 ± 5 in patients with no staining (*p* < 0.05 comparing bilateral staining vs. the other two groups). 

**Figure 2:**
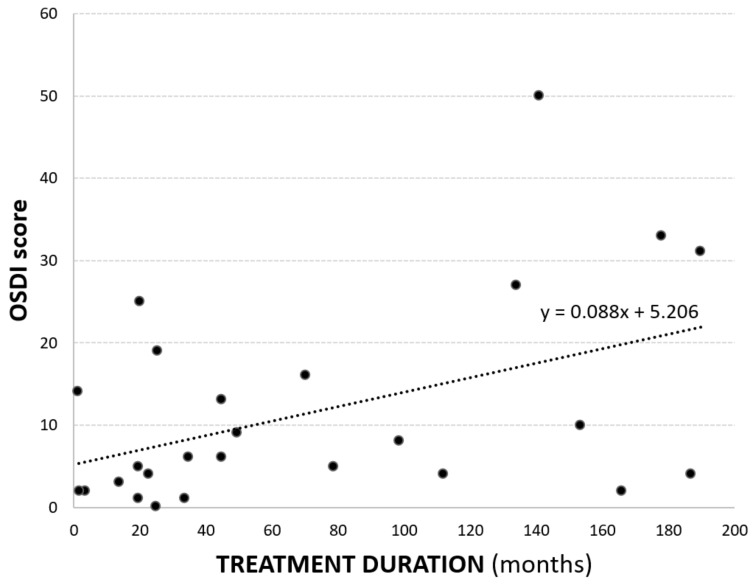
Scatterplot of treatment duration vs. ocular surface disease index (OSDI) score (*r* = 0.45, *p* = 0.03).

The number of molecules used per day, the quantity of preservatives used per day, and the duration of treatment did not significantly correlate with any OS parameter. Correlation was poor with questionnaires too, except for duration of treatment and OSDI score (*p* = 0.03, *r* = 0.45, Figure 2).

## 4. Discussion

This cross-sectional study compared the ocular surface parameters in glaucoma patients with one eye under medical treatment (Med Eyes) and the fellow eye successfully treated with trabeculectomy (Trab Eyes). Overall, Trab eyes showed a better homeostasis of the ocular surface, when assessed by means of tBUT, grading of conjunctival hyperemia and of corneal staining. In the two groups, small but clinically and statistically significant differences were shown. Conversely TFO was not able to detect differences between the two groups, and results were close to the superior interval of normality in most patients, regardless of the presence of mild ocular surface changes as measured with low-tech techniques. Our results are close to Lee at al., who showed mildly elevated TFO in both chronically medicated glaucoma patients as well as in patients with successful trabeculectomy compared with controls [30]. It is possible that TFO may be useful at earlier stages of OSD than those included in our study.

Corneal epithelial damage was the parameter better correlating with questionnaire scores: patients with bilateral staining had significantly worse scores than those with unilateral staining or no staining. This finding is expected if we keep in mind that corneal integrity is essential to good visual acuity, which leads to better questionnaire results. 

In this study we attempted to evaluate the association between ocular surface parameters and medical therapy (number and type of drugs, daily exposure to preservatives, cumulative exposure to preservatives). Apart from a significant deterioration of OSDI score and treatment duration, we failed to show any other direct causal link. This is probably due to the complexity of factors acting on the ocular surface of these patients, to the large differences in treatments and duration of treatments in our cohort of patients, and to the relatively low number of patients included in the study.

The results of this study should be considered with caution due to possible limitations. The number of participants in this study was relatively small, because just a very low minority of patients met inclusion criteria over a very large population of glaucoma patients followed in our hospital. Moreover, we included patients who had received surgery or started medical treatment at any interval from study visit (one patient, though clinically stable, had trabeculectomy 7 months before data collection, and the duration of medical treatment had a very large range from 6 months to 15 years). This may have largely affected our results, as toxic damage is typically dose-dependent [31,32,33]; on the opposite, ocular surface changes due to surgery tend to reduce over time [34]. We also included patients receiving different medical regimes and different exposure to BAK. Finally, the subjectivity and variability of ocular surface parameters should also be considered [25]; in particular, ocular surface parameters are strongly affected by temperature and humidity, and in our setting, it was not possible to control them. A prospective design of such a study could avoid many of these limitations and provide more interesting information in order to analyze ocular surface changes occurring in these patients.

## 5. Conclusions

This study showed that patients successfully treated with medical treatment on one eye and trabeculectomy on the fellow eye show mild ocular surface changes, with trabeculectomy performing overall better than medical treatment when tBUT, conjunctival hyperemia and corneal staining were studied. The presence of corneal epithelial damage in both eyes is the factor more consistently affecting questionnaire scores, and it is also significantly associated with the grading of conjunctival hyperemia.

## Figures and Tables

**Table 1:** Distribution of IOP-lowering treatments in Med Eyes.

Treatments	*n*
Dorzolamide + Timolol FC BID; Bimatoprost OD	6
Dorzolamide + Timolol FC BID; Tafluprost OD	4
Dorzolamide + Timolol FC BID; Travoprost OD	2
Dorzolamide + Timolol FC BID; Bimatoprost OD; Brimonidine BID	1
Dorzolamide + Timolol FC BID	4
Tafluprost OD	1
Brinzolamide + Brimonidine FC BID; Bimatoprost OD	3
Brinzolamide + Brimonidine FC BID; Tafluprost OD	1
Dorzolamide BID; Bimatoprost OD	1
Timolol + Bimatoprost FC OD; Brinzolamide BID	1
Bimatoprost + Timolol FC OD	2

BID, twice daily; FC, fixed combination; OD, once daily.

**Table 2:** Results of questionnaires and ocular surface parameters in the differently treated eyes.

	Trab Eye Mean ± SD	Med Eye Mean ± SD	*p* Value
IDEEL questionnaire	48 ± 38		
OSDI questionnaire	11 ± 12		
TFO (mOsm/L)	307 ± 16	311 ± 22	0.33
Conjunctival hyperemia	0.8 ± 0.9	1.7 ± 1.1	**<0.001**
tBUT (s)	6.5 ± 3.5	5.1 ± 2.7	**0.004**
Positivity at Oxford scale (*n*)	9	15	0.12
Oxford scale	0.3 ± 0.5	0.6 ± 0.5	**0.03**
Schirmer test (mm)	13 ± 9	12 ± 7	0.60

IDEEL, Impact of Dry Eye on Everyday Life; OSDI, Ocular Surface Disease Index; tBUT, tear break-up time; TFO, tear film osmolarity. Bold, *p* < 0.05.

**Table 3:** Distribution of patients showing better parameters in the differently treated eyes.

	Trab Eye Better	No Inter-Eye Difference	Med Eye Better
TFO (*n*)	13	0	13
tBUT (*n*)	17	2	7
Schirmer (*n*)	15	1	10
Conjunctival hyperemia (*n*)	26	0	0
Oxford scale (*n*)	19	7	0

tBUT, tear break-up time; TFO, tear film osmolarity.

## Data Availability

Data will be available upon request to the authors.
